# Australian secondary school principals’, parents’, and students’ attitudes to prescribed school footwear guidelines

**DOI:** 10.1186/s13047-023-00624-0

**Published:** 2023-04-29

**Authors:** Natalie Mazzella, Aaron Fox, Natalie Saunders, Danielle Trowell, Bill Vicenzino, Jason Bonacci

**Affiliations:** 1 Centre for Sports Research, Deakin University, Waurn Ponds, VIC 3215 Australia; 2 Centre for Sports Research, Deakin University, Burwood, VIC 3125 Australia; 3 School of Health and Rehabilitation Sciences, The University of Queensland, St Lucia, QLD 4072 Australia

**Keywords:** Adolescent, Shoes, Footwear, Health, Youth

## Abstract

**Background:**

Adolescents are often required to wear footwear that adheres to uniform guidelines at secondary school. There is a paucity of literature on factors influencing school footwear choice and what drives the development of school footwear guidelines. The aims of this study were to describe (i) current school footwear guidelines in secondary schools across Australia, (ii) factors that influence footwear choice in secondary school students and their parents, and (iii) principals, parents, and students’ beliefs on factors which contribute to school footwear guidelines.

**Methods:**

An online survey was distributed to principals, secondary school students (aged 14–19 years) and their parents across Australia. The survey included questions on current school footwear guidelines, factors influencing footwear choice (for students and parents), participants beliefs on the effect footwear has on musculoskeletal health, current and previous lower limb pain, and beliefs on factors that contribute to school footwear guidelines. Parent and student responses to factors that influence their footwear choice were compared using proportional odds logistic regression. Students and parents’ responses to factors influencing footwear guidelines were compared to principal responses using proportional odds logistic regression. Significance was set at an alpha of < 0.05.

**Results:**

Eighty principals, 153 parents and 120 secondary school students responded to the survey. 96% (77/80) of principals reported that their schools have set guidelines for school footwear. 88% of principals considered comfort to be important when developing school footwear guidelines. Proportional odds logistics regression showed that parents and students were 3.4 and 4.9 times more likely, respectively, than principals to rate comfort as being important when schools develop footwear guidelines. More than 40% of students reported experiencing musculoskeletal pain, and 70% of these students reported the pain to be exacerbated when in their school shoes. Less than a third of participants considered healthcare recommendations important to the development of footwear guidelines.

**Conclusions:**

Nearly all principals that participated in this survey had set guidelines for school footwear. There is a discord between parents, students, and principals on the importance that factors such as comfort, play in the development of school footwear guidelines.

**Supplementary Information:**

The online version contains supplementary material available at 10.1186/s13047-023-00624-0.

## Background

Adolescence is a period of rapid growth and development that has been accompanied with an increased rate of musculoskeletal pain and injury compared to childhood, particularly at the knee and foot [1–3]. Adolescents spend a large percentage of their weekday time at school, where they often must adhere to school uniform guidelines [4, 5]. Anecdotally, secondary school uniform guidelines often require students to dress in formal attire and black, leather shoes, though secondary school footwear requirements have not been documented. School shoes are often designed with stiff, inflexible midsoles, a closed toe and elevated heel heights [6]. Recent evidence has suggested that some of these footwear characteristics increase knee joint loads, reduce tactile feedback during walking and running, and reduce dynamic balance when compared to being barefoot [7–10]. Cross-sectional studies have identified changes in paediatric gait characteristics including step length, velocity, and cadence when children go from being barefoot to shod in school and/or sport shoes [11–14]. No research has yet investigated whether similar changes are apparent in an adolescent cohort when wearing school footwear.

Little is known about the rationale and factors that contribute to school footwear guidelines for adolescents while attending secondary school. Research focused on footwear choice has primarily centred on adults and athletic footwear, with a common belief that footwear is an important factor in the prevention of running related injuries and the management of inflammatory arthropathy [15–17]. Recreational adult runners believe that footwear for children and adolescents should include structural support [18] but there is no current consensus on the best options for school footwear for children and adolescents [19]. Early in adolescence, parents and guardians are highly influential on their dependent’s behaviour [20, 21]. As adolescents mature, peer and friends’ opinions have a greater influence on establishing behavioural norms [21, 22]. Limited research has investigated what drives footwear selection in an adolescent specific cohort. Adolescent cross-country runners report that friends and family are a major source of information influencing their running shoe choice [23]. However, adult footwear knowledge is often influenced by retail footwear stores, popular beliefs, and peers, as opposed to scientific evidence [15, 18]. It is likely that parent and peer opinion are contributors to the selection of school footwear in adolescents. There is a need for greater understanding and consideration of child and adolescent opinions in footwear research [24, 25]. Exploring what secondary school students, as well as parents of secondary school students, consider important when choosing school footwear requires investigation.

Development of school uniform guidelines is often the role of senior heads of school. There is little understanding on the determinants that inform these guidelines. Restrictive uniforms, peer pressure and a lack of school support are reported as barriers to participation in physical activity across adolescence [26–28]. A recent study of principals and parents investigated barriers to school uniform changes in primary schools [29]. Principals were not supportive of a uniform policy that allowed children to wear sports or recreational uniform full time, citing reasons such as school status and cost as barriers to change. In contrast, parents were supportive of the change, indicating a potential discord between parents and principals on school uniform guideline policies. It is not clear if these findings also extend to school footwear recommendations or across secondary schools.

Given the effect that footwear can have on paediatric gait, and the barriers that traditional uniforms pose to physical activity participation [7, 26, 27], identifying the factors most important when selecting footwear for school is needed. Understanding what informs the development of uniform guidelines for school principals also requires investigation as school guidelines are a potential determinant to the selection of school footwear worn by students. Therefore, the aims of this study were to describe (i) current school footwear guidelines in secondary schools across Australia, (ii) factors that influence footwear choice in secondary school students and their parents, and (iii) principals, parents, and students’ beliefs on factors which contribute to school footwear guidelines.

## Methods

### Study design

This study was a cross-sectional anonymous online survey and has been reported using the Checklist for Reporting Results of Internet E-Surveys (CHERRIES) [30]. Ethical approval for this study was granted from the Deakin University Human Research Ethics Committee. Participants were informed of the purpose of the study, the research team involved and the expected completion time of the survey upon opening the survey weblink. Participants were informed that consent was implied upon completion of the survey on the online platform. Participants were advised that participation was voluntary and that they were free to exit the survey at any time. Incomplete responses were excluded from the survey analysis.

### Recruitment

Secondary school students and parents of secondary school students were recruited from all states and territories across Australia from March 2021 to July 2022. Targeted advertising on social media was used in combination with posters placed at shoe stores and local sports medicine clinics. Advertisements were also distributed in school newsletters following approval from the relevant state education department and school principals. Principals were recruited from the contact information listed on their school’s website inviting them to participate in the study.

### Participants

Eligibility criteria for each participant group were as follows: (i) students aged between 14–19 years of age and currently enrolled and attending a secondary school; (ii) parents with a child/children aged between 14–19 years of age currently enrolled and attending a secondary school; (iii) principals employed as a secondary school principal within a government (receive government funding) or non-government school (including catholic and independent schools) [31].

### Survey instrumentation

A survey that was estimated to take 10-min was administered online using Qualtrics (Qualtrics, Provo, United States). Participants completed the survey once, at their own convenience (see Additional file 1). The survey was piloted by the research team and a small number of adolescents (aged 14–19 years) prior to distribution. To protect anonymity of responses, no IP data was collected or stored during the data recording process.

### Survey questionnaire

The survey was comprised of five sections (Additional file 1). Section one collected baseline demographics such as age, gender, and school type. Section two gathered information on current footwear guidelines and footwear requirements for students (such as colour, fixation, material). Section three required respondents to indicate how important the following factors were to footwear choice: shoe comfort, school footwear guidelines, shoe price, opinion of peers and friends, shoe appearance, and healthcare recommendations. A four-point Likert scale was used for participants to respond from not at all important to very important. As secondary school principals are not involved in footwear selection, they did not provide responses to factors influencing footwear choice (section three).

Section four assessed factors influencing the development of guidelines while at secondary school. Factors included foot health, school tradition, comfort, student uniformity, presentation of the school in the community and healthcare recommendations. Principals were asked how important these factors are to the development of their school footwear guidelines. Parents and students were asked how important they believed these factors are to the development of school footwear guidelines at the school they, or their children, attend. Participants responded to this using the same four-point Likert scale. If the participant’s school did not provide guidelines for student footwear, they were exempt from providing responses to this section of the survey. For any schools that did not provide guidelines for footwear, participants were asked to indicate reasons as to why they did not have guidelines.

Finally, students and parents provided responses relating to current or past musculoskeletal pain and the effect that footwear has on their musculoskeletal health and/or pain (section five). Participants were able to indicate multiple sites of pain if applicable. Principals did not provide responses to this section of the survey. At the end of the survey, participants were given the opportunity to add any information using an open-ended response. Participants were able to review their responses prior to submission of the survey.

### Statistical analysis

Survey responses were exported from Qualtrics into Microsoft Excel and RStudio (Version 3.5.2, RStudio Inc, Posit, Boston, United States) for analysis. Descriptive statistics were used to describe the characteristics of the principal, student, and parent responses. Postcode data from respondents was used to classify the school’s location as either urban (major cities) or rural (inner/outer regional/remote) using the Australian Statistical Geography Standard [32] and previously used methodology [29]. To protect anonymity of responses from principals, postcode data for their school was not collected. Participant responses across common items were reported descriptively to determine the frequency of each response.

Responses between parents and students, and between male and female students, on factors that influence their footwear choice were compared using proportional odds logistic regression. The relative importance placed on factors influencing footwear guidelines was also assessed. Students and parents’ responses were compared to principal responses using proportional odds logistic regression. Differences in responses between principals from non-government and government schools were compared using proportional odds logistics regression. Significance was set at an alpha of < 0.05 and odds ratios (95% confidence intervals [CI]) were calculated.

## Results

Four hundred five participants commenced the survey. Of these, 52 responses were excluded due to being incomplete (*n* = 29), and/or being less than 14 years of age (*n* = 21) or being older than 19 years of age for student responses (*n* = 2). 120 secondary school students, 153 parents and 80 secondary school principals completed the survey in full (*n* = 353). Table 1 provides a summary of the demographic characteristics of the participants. Students spent an average of 7.98 [1.95] hours in their school shoes for an average 3 days per week.

**Table 1:** Participant characteristics

Characteristics	n (%)
*Principals*	
All	80 (22.7%)
Female	34 (42.5%)
Male	44 (55.0%)
Prefer not to say	2 (2.5%)
Years in current role	
0–10 years	59 (73.8%)
> 10 years	21 (26.2%)
Sector	
Government	50 (62.5%)
Non-Government	30 (37.5%)
*Students*	
All	120 (34.0%)
Female	75 (62.5%)
Male	42 (35.0%)
Prefer not to say	3 (2.5%)
Age	16.1 (1.48) years
Sector	
Government	64 (53.3%)
Non-Government	56 (46.7%)
Location	
Rural	73 (60.8%)
Urban	47 (39.2%)
*Parents*	
All	153 (43.3%)
Female	143 (93.5%)
Male	10 (6.5%)
Location	
Rural	98 (64.1%)
Urban	55 (35.9%)

### Footwear characteristics

Seventy-seven principals (96%) had set guidelines for school footwear for students. 87% (*n* = 67) of these principals required student’s footwear to be black and include a closed toe box, 73% (*n* = 56) reported a leather shoe was required and 57% (*n* = 44) reported fixation such as laces or buckles was required. There were no differences between principals from government and non-government schools on the type of shoe recommended for school.

### Musculoskeletal health

Fifty-one secondary school students (43%) experienced current pain in their legs or feet, and of these 36 students (71%) reported that their pain was worse when wearing school shoes. 28 students (46%) reported knee pain, 29 students (47%) reported foot and/or leg pain and 4 students (7%) reported dermatological pain (i.e., blisters or ingrown toenails).

### Factors influencing footwear choices

**Fig. 1:**
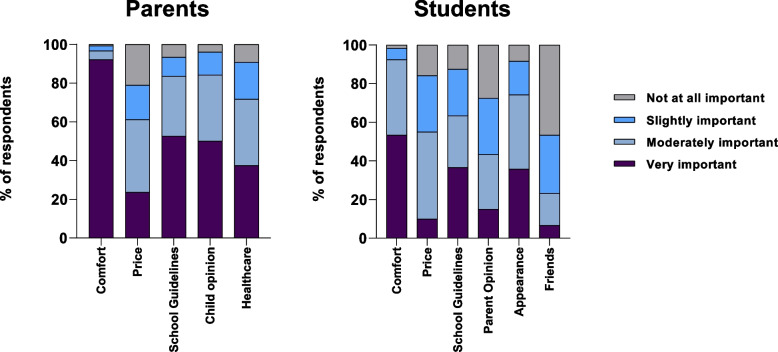
Importance of factors influencing footwear choice of secondary school students and parents of secondary school students

Figure 1 presents the factors influencing footwear choice for secondary school students and parents of secondary school students. 111 students (93%) and 147 parents (96%) reported that comfort was either very important or moderately important to the type of shoe they, or their children, chose to wear to school. 44 students (37%) reported that the school guidelines and the appearance of the shoe were very important to the type of shoe they chose to wear to school. Only eight students (7%) considered their friend’s opinion very important. Female secondary school students were 2.1 [1.0 to 4.2] times as likely to rate appearance as having higher importance to shoe choice when compared to male secondary school students. Female students were 2.0 [1.0 to 4.1] times as likely to rate their parents’ opinion as having a low importance to their shoe choice when compared to male students.

### School footwear guidelines

Of the 353 participants who responded to the survey, 258 (73%) attended, were a principal, or a parent at, a school that had set guidelines for student’s footwear. For the three principals who worked at schools with no footwear guidelines, parental pushback and tradition were cited as barriers to the implementation of guidelines. Twenty-two principals (29%) reported that occupational health and safety requirements were a major determinant of school footwear guidelines within their schools. Shoe cost and socioeconomic status was reported as an additional determinant to school guidelines by five principals (6%).

**Fig. 2:**
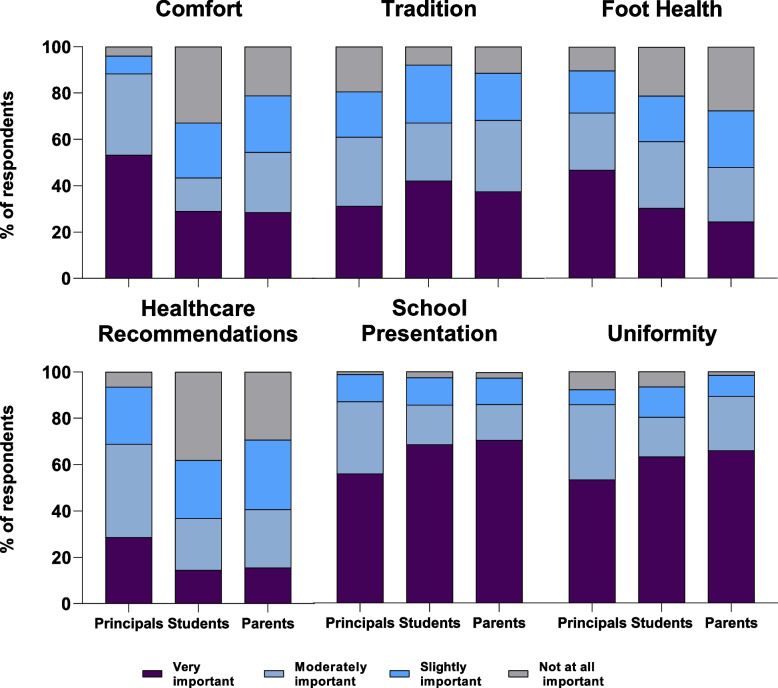
Importance of factors influencing school footwear guidelines for principals, secondary school students and parents of secondary school students

Proportions for participant responses to school footwear guidelines are presented in Fig. 2. Sixty-eight principals (88%) reported that comfort was either very important or moderately important to the development of school footwear guidelines. 33 students (43%) and 67 parents (54%) rated comfort as being important to the developers (i.e., school principals) of school footwear guidelines. Proportional odds logistic regression revealed parents were 3.4 [2.0 to 5.8] and students 4.9 [2.7 to 9.1] times more likely to rate comfort as having lower importance to the development of school footwear guidelines when compared to principals.

Parents were 2.8 [1.7 to 4.7] times and students 3.4 [1.9 to 6.2] times more likely to rate foot health as having lower importance to the development of school footwear guidelines compared to principals. 22 principals (29%) considered healthcare recommendations very important to the development of school footwear guidelines. 19 parents (14%) and 11 students (15%) considered healthcare recommendations important when principals develop school footwear guidelines. Parents were 2.9 [1.7 to 4.9] and students were 3.8 [2.1 to 6.9] times more likely to rate healthcare recommendations as having lower importance to the school guidelines compared to principals.

Principals from non-government schools were 3.5 [1.3 to 9.9] times more likely to rate uniformity as having higher importance to footwear guidelines compared to principals from government schools. Overall, 56 principals (70%) reported that they would consider changing footwear guidelines for students while attending secondary school.

## Discussion

This is the first study to investigate footwear requirements for secondary school students across Australia. Nearly all principals that participated in this study report that their school has guidelines on footwear for students to wear to school. Those principals that did not have guidelines for school footwear were based at a government school. Students spend an average of eight hours per day in their school shoes three days per week. Students are typically expected to wear an all-black closed in shoe with appropriate fixation (i.e., buckles or laces) with no differences in the shoes recommended between government and non-government schools. For the small number of schools that do not provide footwear guidelines, principals report that parental pushback and student resistance are the major barriers to implementation.

Footwear comfort is highly valued by students and parents when selecting the shoes, they, or their children, choose to wear to school. These findings are consistent with studies performed in primary school aged children [8, 25], as well as adult runners [15, 18] and adults with inflammatory arthropathies [17, 33]. Parents of secondary school students consider their child’s opinion very important when selecting shoes. In contrast, more than half of students do not consider their parent’s opinion important. The majority of secondary school students who responded also did not consider the opinion of peers or friends important. This contrasts with previous literature on clothing choice in adolescents which suggests that peer opinion is highly influential on behaviour, particularly in older adolescent cohorts [20, 21]. Consistent with previous research in adolescent clothing choice, female students were twice as likely than male students to value appearance of the shoe important to footwear choice [34]. For students, appearance of the shoe and comfort were the most important factors when selecting the shoes worn to school. The perception of comfort may be influenced by the appearance of the shoe as well as individual physiological factors, gender, wear time and psychosocial and developmental needs [24, 25, 35]. As adolescence is a period of significant physical and psychosocial change, clothing, and by extension footwear, may provide adolescents with an opportunity to express their identity [36] which may go some way to explaining the high value placed on appearance by our cohort.

Less than a quarter of parents and students feel that foot health is considered important when principals, or heads of school, design school footwear guidelines. However, our findings suggest that principals do believe these factors, along with occupational health and safety requirements, are important when designing footwear guidelines. Most parents and students believe that principal’s place greater importance on student uniformity and tradition when recommending school footwear for students. Previous research has demonstrated similar findings between principals and parents [29]. Parents of primary school students were highly supportive of students wearing sports uniform daily in comparison to principals, who placed greater emphasis on school presentation. In our study principal’s report that they consider foot health, comfort, and presentation of the school important however, there is likely some differences in which factors take priority when developing school footwear guidelines. It is not clear whether our findings would have differed if participants were required to rank the factors in order of importance to footwear guideline development. Considering that 63% of students and 84% of parents indicated that school guidelines are important to footwear choice, a greater understanding of footwear guideline development may better support their decision making.

Lower limb pain was commonly reported by participants in this survey, specifically at the knee and foot. Musculoskeletal pain of the lower limb is frequent in adolescents [3] and, according to the results of this study, may be influenced by shoes worn to school. Shoes are considered important to the prevention of running related injuries by adults [15]. Running biomechanics, foot function, and balance can be influenced by shoes worn in adolescents and children [7, 9]. Footwear with elevated heel heights and stiff midsoles have been shown to elevate knee joint loading in adults and adults with anterior knee pain [37, 38]. However, there is no prospective evidence that demonstrates the effect that footwear has on pain and/or injury risk in an adolescent cohort [39]. Recommendations from healthcare practitioners were not considered very important by most participants included in this survey. Considering that school shoes are the most common shoe worn by adolescents during the school week, identifying the effect that recommended school footwear may have on musculoskeletal health and lower limb biomechanics is needed.

### Limitations

There were limitations of this study that require acknowledgement. Firstly, we did not collect responses from adolescents less than 14 years of age. This was due to the nature of the survey being online and anonymous. Adolescents aged 14 and above were included as they were deemed mature enough to understand the questions and respond appropriately. It is possible that younger adolescents may have different perceptions on the factors which influence their choice of school shoe that are not captured within this research. Secondly, we did not collect data on the specific type of shoe currently worn by students experiencing musculoskeletal pain in this study. This was due to the lack of consistent taxonomy in paediatric footwear at the time of survey distribution. Recent work has highlighted gaps in agreed footwear taxonomy for children (less than six years of age) [40]. The same definitions for footwear styles was not applicable to school footwear and the population of interest in this study. Although secondary school students reported musculoskeletal pain while wearing school footwear, an examination of which type or style of school shoe was associated with pain was not possible. Future research may look to examine this. Finally, our sample only included data from students, parents, and principals from Australia. It is possible that perceptions relating to school footwear may differ across different cultures and countries. Similarly, selection bias is plausible as recruitment for this study was primarily performed across social media channels which may predispose similar cohorts of parents and students to participation or those already interested in this topic to respond. We had a slightly higher proportion of responses from students and principals from government schools. As our results indicate no difference in the type of shoe recommended for school between government and non-government schools, the effect of a larger response from participants from government schools is not clear.

## Conclusion

Nearly all secondary school principals that participated in this survey required their students to wear an all-black, closed toed shoe for school. When designing these recommendations, principals report that they consider student comfort, foot health, safety, and school presentation to be highly important. In contrast, secondary school students and their parents believe that school tradition and student uniformity have higher importance to school footwear guidelines. Students and parents highly value comfort and school guidelines over shoe cost when choosing footwear worn to school. Greater communication from principals and schools on factors influencing their footwear guidelines may alleviate students and parents’ concerns relating to factors such as foot health and comfort.

## Supplementary Information

**Additional file 1: Appendix 1.** Survey questionnaire.

## Data Availability

Data from this survey is non-identifiable. Responses are anonymous with no identifying information collected nor any email or IP address information.

## Abbreviation

CI: Confidence interval
