# Supplementary material for: Australian secondary school principals’, parents’, and students’ attitudes to prescribed school footwear guidelines
Source: J Foot Ankle Res. 2023 Apr 29;16:26. doi: 10.1186/s13047-023-00624-0 (PMC10148548; doi:10.1186/s13047-023-00624-0)
Supplement: Supplementary file 1 — Additional file 1: Appendix 1. Survey questionnaire. [file 13047_2023_624_MOESM1_ESM.pdf]

Appendices & Supplementary Material

Appendix 1: Survey questionnaire

Australian secondary school principals, parents and students attitudes to prescribed school footwear guidelines

Start of Block: Plain Language Statement inserted

- ○ I consent to participate in this survey
- ○ I do not consent to participate in this survey

Q1.2 I am a:

- ○ Secondary school principal
- ○ Parent or guardian of a secondary school student/s
- ○ Secondary school student (Aged 14-19)

Start of Block: Principal Demographics

Q2.1 The following set of questions relate to baseline information about you and the secondary school you work within.

Q2.2 What is your gender?

- ○ Male
- ○ Female
- ○ Prefer not to say

Q2.3 What is the length of time in your current role?

- ○ 0-5 years
- ○ 6-10 years
- ○ 11-15 years
- ○ 15+ years

Q2.4 How many years have you been working within a school setting?

- ◯ 0-5 years
- ◯ 6-10 years
- ◯ 11-15 years
- ◯ 15+ years

Q2.5 What type of school setting do you work within?

- ◯ Government (Public), CoEd
- ◯ Government (Public), Male only
- ◯ Government (Public), Female only
- ◯ Non Government (Private, Independent, Catholic), CoEd
- ◯ Non Government (Private, Independent, Catholic), Male only
- ◯ Non Government(Private, Independent, Catholic), Female only

---

Q2.6 What state is your school in?

- ◯ Victoria
- ◯ New South Wales
- ◯ Queensland

End of Block: Principal Demographics

Start of Block: School Footwear Guidelines

Q3.1 The following questions ask what your school (or you as principal) recommend your students to wear on their feet.

Q3.2 Does your school provide guidelines on what footwear needs to be worn by students at school?

- ◯ Yes
- ◯ No

Q3.3 Please select any reasons why your school does not provide guidelines or recommendations for footwear to be worn by students.

- ▢ Longstanding tradition
- ▢ Parental pushback
- ▢ Student resistance
- ▢ Current uniform policy
- ▢ Too controversial
- ▢ Unclear on what should be recommended
- ▢ ⊗No specific reason
- ▢ Other ____

Q3.4 Does your school recommend a specific brand of footwear for students to wear? If yes, please list the name/s of the recommended brands

- ○ No
- ○ Yes ____

Q3.5 Does your school provide or outline any of the following requirements for school footwear? (Select all that apply)

- ▢ Shoe must be black
- ▢ Shoe must be leather
- ▢ Shoe must use fixation such as laces, buckles or velcro
- ▢ Shoe must have a specific heel height. If yes, please comment on the height in centimetres (cm) ____
- ▢ Shoe must not be slip on
- ▢ Shoe must be closed in at the toe
- ▢ Other ____

Q3.6 How much importance do the following factors have on the development of guidelines for school footwear for students to wear each day?

	Not at all important	Slightly important	Moderately important	Very important
Promotion of good foot and leg health	○	○	○	○
School tradition	○	○	○	○
Student comfort	○	○	○	○
Student uniformity	○	○	○	○
Presentation of the school in the community	○	○	○	○
Recommendations provided by health professionals	○	○	○	○

Q3.7 Would your school be open to changing the uniform guidelines relating to footwear?

- ◯ No
- ◯ Maybe
- ◯ Yes

End of Block: School Footwear Guidelines

Start of Block: Factors Influencing School Footwear Guidelines & Conclusion

Q4.1 The following questions are designed to help us to understand any further areas that may contribute to school footwear recommendations.

Q4.2 Please rate how you feel footwear impacts musculoskeletal and lower limb health.

- ◯ Not at all important
- ◯ Slightly important
- ◯ Moderately important
- ◯ Very important

Q4.3 Is there anything else you would like to add that you feel we have missed in relation to school footwear recommended by you or your school for students?

____

____

____

____

____

End of Block: Conclusion

Start of Block: Student Demographics

Q5.1The following questions ask about you and the type of school you attend

---

Q5.2 What is your age (in years)?

____

Q5.3 What is your sex?

○ Male

○ Female

○ Prefer not to say

Q5.4 What state is your school located in?

○ Victoria

○ New South Wales

○ Queensland

Q5.5 What postcode or suburb is your school located in?

____

Q5.6 What type of school do you attend?

○ Government (Public), CoEd

○ Government (Public), Male only

○ Government (Public), Female only

○ Non Government (Private, Independent, Catholic), CoEd

○ Non Government (Private, Independent, Catholic), Male only

○ Non Government(Private, Independent, Catholic), Female only

End of Block: Student Demographics

Start of Block: Footwear Characteristics

Q6.1 The following questions are about the shoes your school recommends for you to wear to school each day

Q6.2 On average, how many hours a day do you spend in your school shoes?

____

Q6.3 How many days per week do you wear sports uniform or athletic footwear to school?

____

Q6.4 How many years have you worn the style of school shoe you currently wear to school?

- ○ Less than one year
- ○ 1-2 years
- ○ 3-4 years
- ○ 4 or more years

Q6.5 Over the last week, how many days did you participate in any form of physical activity or sport (including walking) for more than 10 minutes during the lunch break in your school shoes?

____

End of Block: Footwear Characteristics

Start of Block: Choosing Footwear

Q7.1 The following section will ask why you choose the shoes you wear each day to school.

Q7.2 How important are the following factors on your choice of shoes you wear to school each day?

	Not at all important	Slightly important	Moderately important	Very important
Comfort of the shoe	◯	◯	◯	◯
What your friends and peers wear	◯	◯	◯	◯
The school's uniform guidelines	◯	◯	◯	◯
Your parent or guardian's opinion	◯	◯	◯	◯
Appearance of the shoe	◯	◯	◯	◯
Price of the shoe	◯	◯	◯	◯

End of Block: Choosing Footwear

Start of Block: Factors Influencing School Footwear Guidelines

Q8.1 The following section will ask you about your school's guidelines the shoes you are expected to wear to school.

Q8.2 Does your school provide recommendations on shoes for students to wear to school?

◯ Yes

◯ No

Q8.3 How much importance do you feel the school places on the following factors when recommending the shoe you should wear to school? For example, how important do you feel school tradition is to the school when recommending shoes for students.

	Not at all important	Slightly important	Moderately important	Very important
Good foot and leg health	○	○	○	○
School tradition	○	○	○	○
Your comfort when wearing the shoes	○	○	○	○
Student uniformity	○	○	○	○
Presentation of the school in the community	○	○	○	○
Recommendations provided by health professionals	○	○	○	○

End of Block: Factors Influencing School Footwear Guidelines

Start of Block: Health of your muscles, joints and bones

Q9.1 In this section, you will be asked questions about any injuries or pain you have experienced in your lower limb (such as your ankle, knee, shin or foot). You should think about any pain or injury that you currently have or have had in the past 12 months.

Q9.2 Do you have any current pain (such as aching or discomfort) in your legs or feet? If yes, please list where you experience this pain.

○ No

○ Yes ____

Q9.3 Do your legs (such as your knees, shins or ankle) or feet get sore when wearing your school shoes?

○ No

○ Yes ____

Q9.4 What impact do the shoes you wear to school have on the pain in your legs and feet?

- A great deal worse
- Moderately worse
- A little bit worse
- None at all
- A little bit better
- Moderately better
- A great deal better

Q9.5 How important do you believe footwear is on your joint, muscle and bone health?

- Not at all important
- Slightly important
- Moderately important
- Very important

Q9.6 Out of the options provided, please select the shoe you would prefer to wear to school (Pick one selection only)

- Leather Lace Up or Buckled Shoe
- Flexible Lace Up or Flexible Buckled Shoe
- Runner or Sports Shoe

Q9.7 Is there anything you think we should consider about your footwear that we have not asked in this survey?

____

____

____

____

____

End of Block: Health of your muscles, joints and bones

Start of Block: Parent Demographics

Q10.1 The following set of questions relate to you

Q10.2 What is your age (in years)?

____

Q10.3 What is your gender?

- ○ Male
- ○ Female
- ○ Prefer not to say

Q10.4 How many children do you have enrolled in secondary school?

- ○ 1
- ○ 2
- ○ 3
- ○ 4
- ○ 5
- ○ 6
- ○ 7
- ○ 8

End of Block: Parent Demographics

Start of Block: Child/Children Demographics

Q11.1 The following set of questions relate to your child / children and the type of school they attend. If you have multiple children, please answer the questions in age order. For example, the first set of questions will relate to your eldest child and then descending order from then on.

Q11.2 What age is your child/children?

- ◯ 11 years of age
- ◯ 12 years of age
- ◯ 13 years of age
- ◯ 14 years of age
- ◯ 15 years of age
- ◯ 16 years of age
- ◯ 17 years of age
- ◯ 18 years of age
- ◯ 19 years of age

Q11.3 What is your child/children's gender?

- ◯ Male
- ◯ Female
- ◯ Non-binary / third gender
- ◯ Prefer not to say

Q11.4 What year level is your child/children completing?

- ◯ Year 7
- ◯ Year 8
- ◯ Year 9
- ◯ Year 10
- ◯ Year 11
- ◯ Year 12

Q11.5 What state is your child/children's school in?

- ○ Victoria
- ○ New South Wales
- ○ Queensland

Q11.6 What postcode is your child/children's school located in?

____

End of Block: Child/Children Demographics

Start of Block: Footwear characteristics

Q12.1 The following questions relate to the type of shoe your child/children wear to school. If your child wears more than one type of shoe to school, please answer the questions based on the shoe recommended by their school.

Q12.2 On average, how often does your child/children update their footwear for school?

- ○ More than once a year
- ○ Once a year
- ○ Every two years or more

Q12.3 When purchasing school shoes, does your child/children have their shoes fitted at a store?

- ○ Yes
- ○ Sometimes
- ○ No

Q12.4 Does your child/children have a specific style or brand of shoe they like to wear for school? If yes, please indicate the preferred brand below.

- ○ No
- ○ Yes ____

Q12.5 How important are the following factors on the choice of shoe your child/children wear to school each day.

	Not at all important	Slightly important	Moderately important	Very important
Child/children's opinion	○	○	○	○
Price of the shoe	○	○	○	○
Child/children's comfort	○	○	○	○
Recommendations provided by health professionals	○	○	○	○
School guidelines	○	○	○	○

End of Block: Footwear characteristics

Start of Block: Factors influencing footwear guidelines

Q13.1 The following questions relate to factors influencing the development of guidelines and policies for school footwear to be worn by students.

Q13.2 Does your child/children's school provide recommendations on shoes for students to wear to school?

○ Yes

○ Unsure

○ No

Q13.3 Do you feel the school your child/children attend provide flexibility on which shoes need to be worn to school each day? If yes, please comment on any reasons provided by the school to wear alternative footwear.

○ No

○ Yes ____

Q13.4 Are you satisfied with the recommendations for footwear set out by your child/children's school?

○ Not satisfied

○ Somewhat satisfied

○ Satisfied

Q13.5 How much importance do you feel the school places on the following factors when recommending the shoe your child/children should wear to school? For example, how important do you feel school tradition is to the school when recommending shoes for students.

	Not at all important	Slightly important	Moderately important	Very important
Good foot and leg health	○	○	○	○
School tradition	○	○	○	○
Student comfort	○	○	○	○
Student uniformity	○	○	○	○
Presentation of the school in the community	○	○	○	○
Recommendations provided by health professionals	○	○	○	○

End of Block: Factors influencing footwear guidelines

Start of Block: Health of muscles, joints and bones

Q14.1 The following set of questions relates to any injuries or pain that your child experiences or has experienced in their lower limb such as their ankle, knee, shin or foot. You should think about any pain or injury that they currently have or have had in the past 12 months.

Q14.2 Does your child/children have any pain in their legs or feet at the moment? If yes, please list the areas where the pain is located.

○ No

○ Yes ____

Q14.3 Has your child/children previously reported any pain in their legs or feet while at secondary school? If yes, please list the areas where the pain is located.

- ○ No
- ○ Yes ____

Q14.4 What impact did the shoes your child/children wear to school have on the pain in their legs and feet?

- ○ A great deal worse
- ○ Moderately worse
- ○ A little bit worse
- ○ None at all
- ○ A little bit better
- ○ Moderately better
- ○ A great deal better

Q14.5 What impact do the shoes your child/children wear to school have on the pain in their legs and feet?

- ○ A great deal worse
- ○ Moderately worse
- ○ A little bit worse
- ○ None at all
- ○ A little bit better
- ○ Moderately better
- ○ A great deal better

Q14.6 Did you seek guidance or treatment for your child/children from a health professional (doctor, physiotherapist, osteopath etc) for this pain?

○ No

○ Yes

Q14.7 Have you sought guidance or treatment for your child/children from a health professional (doctor, physiotherapist, osteopath etc) for this pain?

○ No

○ Yes

Q14.8 How important do you believe footwear is on your child/children's joint, muscle and bone health?

○ Not at all important

○ Slightly important

○ Moderately important

○ Very important

Q14.9 Out of the options provided, please select the shoe you would prefer your child/children to wear to school (Pick one selection only)

○ Leather Lace Up or Buckled Shoe

○ Flexible Lace Up or Flexible Buckled Shoe

○ Runner or Sport Shoe

Q14.10 Is there anything you think we should consider about your child/children's footwear that we have not asked in this survey?

____

____

____

____

____

End of Block: Health of muscles, joints and bones
